# Quantitative Assessment of the Learning Curve for Robotic Thyroid Surgery

**DOI:** 10.3390/jcm8030402

**Published:** 2019-03-22

**Authors:** HyunGoo Kim, Hyungju Kwon, Woosung Lim, Byung-In Moon, Nam Sun Paik

**Affiliations:** Department of Surgery, Ewha Womans University Medical Center, 1071 Anyangcheon-ro, Yangcheon-Gu, Seoul 07985, Korea; kara8011@naver.com (H.K.); limw@ewha.ac.kr (W.L.); mbit@ewha.ac.kr (B.-I.M.); namsun.paik@gmail.com (N.S.P.)

**Keywords:** learning curve, CUSUM, robotic, thyroid

## Abstract

With the increased utilization of robot thyroidectomy in recent years, surgical proficiency is the paramount consideration. However, there is no single perfect or ideal method for measuring surgical proficiency. In this study, we evaluated the learning curve of robotic thyroidectomy using various parameters. A total of 172 robotic total thyroidectomies were performed by a single surgeon between March 2014 and February 2018. Cumulative summation analysis revealed that it took 50 cases for the surgeon to significantly improve the operation time. Mean operation time was significantly shorter in the group that included the 51st to the 172nd case, than in the group that included only the first 50 cases (132.8 ± 27.7 min vs. 166.9 ± 29.5 min; *p* < 0.001). On the other hand, the surgeon was competent after the 75th case when postoperative transient hypoparathyroidism was used as the outcome measure. The incidence of hypoparathyroidism gradually decreased from 52.0%, for the first 75 cases, to 40.2% after the 76th case. These results indicated that the criteria used to assess proficiency greatly influenced the interpretation of the learning curve. Incorporation of the operation time, complications, and oncologic outcomes should be considered in learning curve assessment.

## 1. Introduction

Robotic thyroidectomy has been described as an alternative method for removing the thyroid gland without incising the neck. The bilateral axillo-breast approach (BABA) is one of the more popular techniques for robotic thyroidectomy [1]. Among the various remote-access approaches, BABA has several advantages [2]. BABA provides a symmetrical operative view, which is similar to that of open thyroidectomy. This midline approach allows optimal visualization and dissection for vital structures in both thyroid lobes. Moreover, BABA affords the largest operative angles for instrument insertion and manipulation, which can minimize instrument fighting or crowding. Evidence of the cosmetic superiority and surgical safety of BABA robotic thyroidectomy has also been widely reported [3,4,5]. With the increased utilization of robot thyroidectomy in recent years, surgeon training and proficiency are paramount considerations.

The relationship between patient outcomes and surgeon experience has been extensively investigated over the last 10 years [6]. Many studies have demonstrated that more experienced surgeons are associated with decreased operation times and complication rates. Learning curves are commonly used to plot the number of cases necessary to acquire robotic skills and gain the mastery and proficiency of an experienced surgeon [7]. Previous studies evaluating the learning curve for robotic thyroid surgery indicated that operation times have been shown to decrease gradually and reach a steady state only after 35–40 cases are performed by an individual surgeon [8,9,10]. However, these studies have used only operation time as the factor for determining the learning curve. Operation time may not be the most appropriate marker of learning, as speed does not equate to proficiency.

A recent systematic review indicated that learning curves for oncologic surgery could be analyzed using various outcomes [6]. These outcomes are classified into six main categories: operation times, intraoperative outcomes, postoperative outcomes, complications, oncologic outcomes, and surgical success outcomes. Of these categories, operation time was the most commonly used outcome because it is easily measured and compared [7,11]. However, learning curves should also refer to other important indicators for patient care, including functional or oncologic outcomes. As postoperative hypoparathyroidism is the most common complication after total thyroidectomy, we set the functional outcome variable as the incidence of transient hypoparathyroidism. The present study evaluated the learning curve for BABA robotic thyroidectomy using operation time and postoperative transient hypoparathyroidism.

## 2. Materials and Methods

### 2.1. Study Population

Our institutional review board approved this retrospective study and waived the requirement for written informed consent (Approval No. 2017-09-057). This study included 172 consecutive patients with thyroid cancer who underwent BABA robotic total thyroidectomy from March 2014 to February 2018. The BABA technique used in this series is described elsewhere [12]. The operative indications for BABA robotic thyroidectomy in my institution included tumor size under 2 cm, tumors without gross extrathyroidal extension, and no lymph node metastasis on ultrasound. Ipsilateral prophylactic central lymph node dissection was performed in all cases. All surgeries were performed by a single surgeon (H. Kwon), who had little prior experience with endoscopic thyroid surgery. The vocal cords were examined routinely 1 day before and 2 weeks after the thyroidectomy using video-assisted laryngoscopy. Serum concentrations of parathyroid hormone, calcium, phosphorus, and ionized calcium were measured 1 day before and 2 days after the surgery. All patients were followed at intervals of 2 weeks, 3 months, and then 6 months. Follow-up tests included clinical evaluation for hypocalcemia, vocal cord integrity, and thyroid function. Demographic data, pathologic stage, operation time, operative complications, postoperative serum thyroglobulin level, and remnant thyroid tissue on ultrasound were recorded and analyzed.

### 2.2. Definitions

Operation time was defined as the time from skin flap elevation to the detachment of the robot from the patient. Transient hypoparathyroidism was defined as a postoperative serum calcium level <8.5 mg/dL (normal range, 8.5–10.2 mg/dL) with a subnormal parathyroid hormone level (<15 pg/mL). Permanent hypoparathyroidism was defined as a requirement for calcium or vitamin D supplementation for periods >6 months, following surgery. Recurrent laryngeal nerve (RLN) injury, defined as a postoperative laryngoscopic impairment of the motility of the vocal cords, was considered permanent in patients exhibiting persistent impairment at 6 months after surgery.

### 2.3. Statistical Analysis

Cumulative summation (CUSUM) analysis was designed for the quantitative estimation of the learning curve. The CUSUM test shows the cumulative differences between the target data and the observed data. This method was used to plot the operation time and determine the learning curve for operation time [13]. Briefly, the 172 cases were ordered chronologically, from the earliest to the latest surgery date. The difference between the operation time of the *i*th case and the mean operation time was defined as S*_i_*. The S*_i_* values were sequentially summed and then plotted graphically using the equation CUSUM = ΣS*_i_* [14].

The learning curve for transient hypoparathyroidism was assessed using the learning curve CUSUM (LC-CUSUM) and standard CUSUM methods [15]. The LC-CUSUM test computes a score from successive outcomes; an operation without complications increases the score, and an operation with complications decreases the score. The LC-CUSUM method is helpful in determining whether a procedure or proficiency has reached an acceptable level. After achieving surgical competence, we applied the standard CUSUM to ensure the complication rate did not deviate from optimal performance. A more detailed description of LC-CUSUM and standard CUSUM with regards to the formulation, performance, and limits, is provided elsewhere [15,16,17]. Since the two largest series including over 1000 patients showed transient hypoparathyroidism rates of 48.1% and 39.1%, the unacceptable failure rate was set as 0.5 (transient hypoparathyroidism occurring in 50% of patients) during the learning curve period in the present study [18,19]. We also chose an acceptable failure rate of 0.4 and a control limit of 1.25, accordingly. During the control period using the standard CUSUM, the target failure rate was set at 0.6 with a control limit of 2.5.

We used SPSS version 22.0 (IBM Corp., Armonk, NY, USA) for all statistical analyses. Continuous data were compared using Student’s *t*-tests and dichotomous data were compared using chi-squared tests. Correlation coefficients (*R*) were calculated using bivariate correlation analysis. A *p*-value less than 0.05 was considered statistically significant.

## 3. Results

The characteristics of the included patients are summarized in Table 1. BABA robotic total thyroidectomy was successful in all patients, and none required conversion to conventional open surgery. The mean operation time was 105.4 ± 30.3 min (range, 36–200). The mean number of retrieved lymph nodes was 5.4 ± 4.4, and the mean weight of resected thyroid glands was 21.0 ± 8.5 g. Seventy-eight patients (45.3%) experienced postoperative hypoparathyroidism, including 2 (1.2%) patients with permanent hypoparathyroidism. Nine patients (5.2%) experienced postoperative RLN palsy, but no patients exhibited RLN palsy upon laryngoscopic exam after 3 postoperative months. The mean postoperative suppressed thyroglobulin level at 3 months was 0.18 ± 4.4 ng/mL, with 98.3% of patients having thyroglobulin levels of <1.0 ng/mL. Neck ultrasound at 6 postoperative months showed no remnant thyroid tissue in all patients.

**Figure 1:**
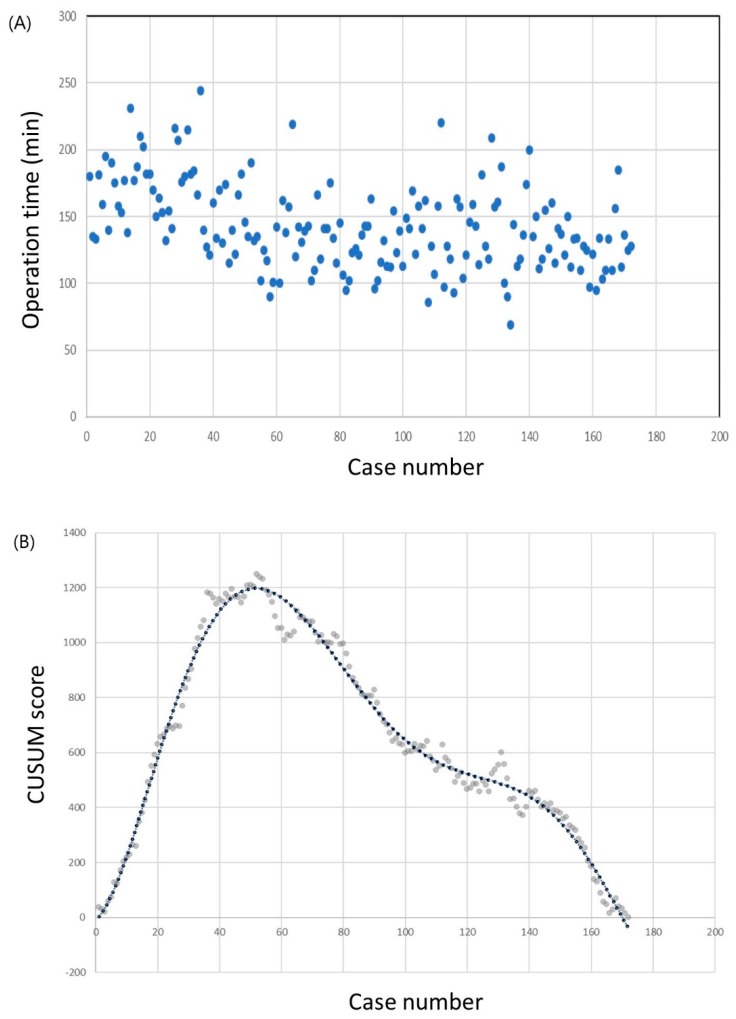
Operation time of the robotic thyroid surgery. (**a**) Operation time plotted in chronological order. (**b**) Cumulative summation test of operation time.

The operation times and CUSUM learning curves are plotted in Figure 1. The mean operation time was 142.7 ± 32.1 min. The best fit for the curve was a sixth-order polynomial with the equation

CUSUM = −6.8908 + 9.1341 × (Case number) + 1.8883 × (Case number)^2^ − 0.0542 × (Case number)^3^ + 0.0006 × (Case number)^4^ − 3 × 10^−6^ × (Case number)^5^ + 5 × 10^−9^ × (Case number)^6^,

which had a high *R*^2^ value of 0.983. The slope of the learning curve turned from positive to negative after the 50th case, which means that the surgeon required 50 cases to achieve mastery. The mean operation time was significantly shorter in the group that included the 51st to the 172nd case than in the group that included only the first 50 cases (98.0 ± 27.6 min vs. 123.5 ± 29.3 min; *p* < 0.001). Other clinicopathological factors, including complication rates, showed no differences between the groups (Table 2).

**Figure 2:**
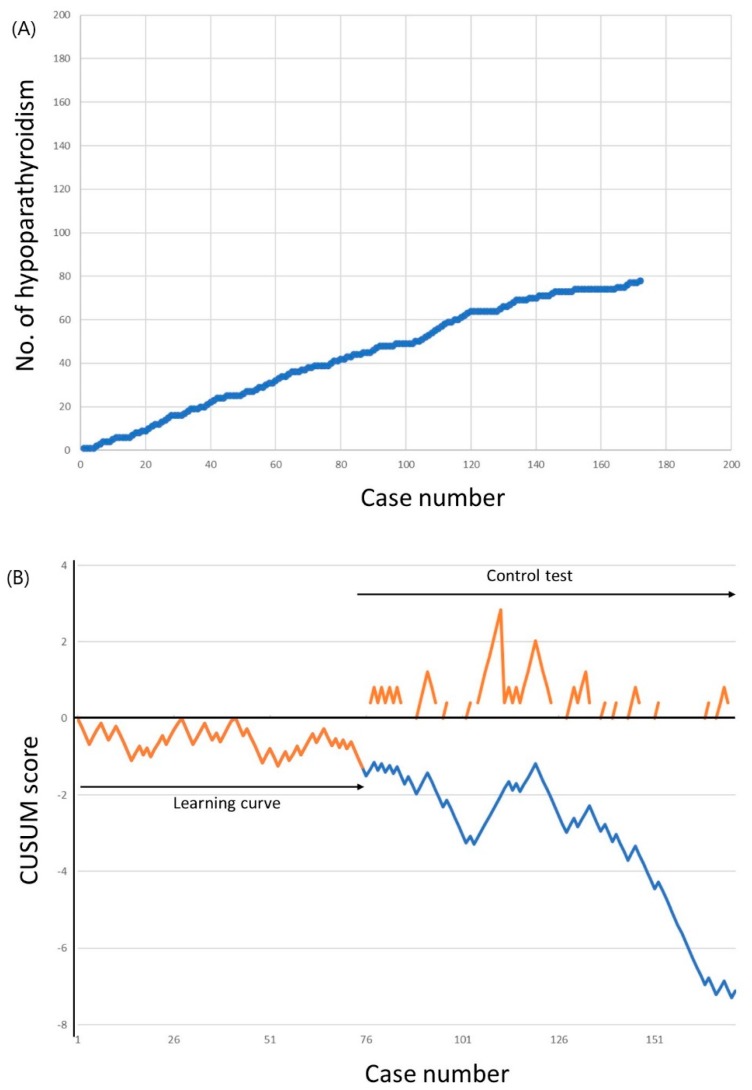
Incidence of transient hypoparathyroidism. (**a**) Cumulative number of transient hypoparathyroidism. (**b**) Cumulative summation test of the hypoparathyroidism incidence for learning curve (LC-CUSUM) and standard cumulative summation (CUSUM) test. The dotted line represents the LC-CUSUM as if it had been continued after the 75th case.

The cumulative sums of transient hypoparathyroidism incidence are shown in Figure 2. The incidence of hypoparathyroidism was 52.0% for the first 50 cases, which decreased to 46.0% for the 51st to the 100th case, and to 40.3% for the 101st to the 172nd case. The LC-CUSUM analysis indicated that the surgeon was proficient at the 75th case. Until this point, 39 patients (52.0%) experienced transient hypoparathyroidism, which was above the unacceptable failure rate cutoff of 50%. However, from the 66th to the 75th case, the incidence of hypoparathyroidism was 30.0%, which was below the acceptable failure rate of 40.0%. The learning curve for minimizing transient hypoparathyroidism, therefore, required 75 cases. A standard CUSUM analysis was started after the 75th case to ensure that the surgeon did not deviate from ideal performance. Although the alarm was raised after case number 111, no further alarm was raised to the end of 172 completed cases.

As the LC-CUSUM analysis could be threshold-dependent, we further performed sensitivity analyses (Table 3). The learning curve for minimizing transient hypoparathyroidism varied widely from 4 to 163 cases, depending on the different threshold levels. Most of the learning curves in the sensitivity analyses ranged from 73 to 76 cases, although lower complication rates needed more cases for proficiency. The learning curve of 75 cases in the present study—using an unacceptable failure rate of 50.0% and an acceptable failure rate of 40.0% with decision limit 1.25—was also comparable with these sensitivity analyses.

## 4. Discussion

This study demonstrated that a surgeon required more completed operations to achieve proficiency at BABA robotic thyroidectomy when the incidence of transient hypoparathyroidism was used as the marker of proficiency, as opposed to operation time being used as the proficiency marker. Our findings also indicated that learning curve assessments should consider several variables, including functional or oncologic outcomes. In the field of robotic surgery, significant interest has been placed on surgeon performance evaluations, and the emphasis on safety and outcomes. Less than half of the previously published research on this topic, however, employed proper methods and appropriate outcome measures [7]. Qualitative or subjective methods cannot formally or impartially evaluate surgical competence, and their reproducibility and objectivity are questionable [20]. Studies specifically investigating the learning curve of robotic thyroidectomy have had similar limitations: nonobjective methods without statistical modeling or stratification and use of operation time as the only outcome measure [8,9,10]. To overcome these problems, we applied statistical modeling and compared learning curves that used operation time and postoperative hypoparathyroidism as markers of proficiency.

Observation by a tutor and graphical description of learning curves have been the most common ways of judging individual performance; however, both are prone to subjectivity [20,21]. Objective clinical performance can be measured using statistical methods, including the Shewhart p-chart, g-chart, exponentially weighted moving average chart, and CUSUM chart [22]. Among all of these methods, CUSUM has gained popularity and has been widely disseminated due to its simple formulation, its capability to detect small persistent changes, and its intuitive depiction [23]. The CUSUM method has also shown its capacity for detecting fatal errors, near misses, and suboptimal performance in a timely fashion [23]. We used LC-CUSUM, which was developed to enable quantitative assessment of individual performance and signals when a predefined level of performance has been achieved [15].

There is no single perfect or ideal method to measure surgical proficiency. However, learning curve evaluations should incorporate operation time, indicators of complications, and indicators of success [6]. Although we included operation time and a complication indicator in our analysis, no indicator of success was investigated in this study. Possible indicators of success for robotic thyroidectomy include postoperative serum thyroglobulin levels, remnant thyroid tissue on ultrasound, and recurrence of thyroid cancer. Monitoring of these factors, however, may be associated with poor statistical properties because of the limited number of expected events. In the present study, suppressed thyroglobulin levels at 3 postoperative months was <1.0 ng/mL in 98.3% of patients. Furthermore, no remnant thyroid was found on ultrasound after robotic thyroidectomy in all patients. Accordingly, we would not have been able to incorporate these indicators of success in the present analysis.

A recent meta-analysis reported that the median incidence of postoperative transient hypoparathyroidism ranged from 19.0% to 38.0% after conventional open thyroidectomy [24]. This incidence is comparable to that of robotic thyroid surgery, which has been shown to range from 39.1% to 48.1% [18,19]. The causes of postoperative hypocalcemia include disruption of the parathyroid arterial supply or venous drainage, thermal or electrical injury, mechanical injury, and either intentional or inadvertent removal [25]. As the parathyroid blood supply is both delicate and complex, close attention and experienced surgeons are required to ensure its preservation and to prevent hypoparathyroidism [7,26]. An analysis of the US National Inpatient Sample data also found an association between higher surgeon experience and lower hypoparathyroidism rates [27,28]. The incidence of transient hypoparathyroidism, therefore, was used as the surrogate marker of complications for learning curve evaluation. Our results indicated that at least 75 cases of BABA robotic thyroidectomy might be needed to overcome the learning curve.

This study had some limitations. First, the learning curve data were derived from the robotic thyroidectomy procedures of only one surgeon. For LC-CUSUM analysis, there was an assumption that each observation had no serial correlation [17]. Although we tried not to violate this assumption, a single novice operator had potentials for serial correlation. Furthermore, learning curves may also vary between individual surgeons based on experience in laparoscopy, surgical skills, or familiarity with the procedure [29]. This can limit the external validity of our findings, and generalization of the results should be made with caution. Second, the acceptable complication rates were relatively high. In the LC-CUSUM analysis, the adequate and inadequate levels for performance can be arbitrarily chosen by consensus. Although we used the complication rates from the two largest series of over 1000 patients, the acceptable level can be lower in the future. Third, learning curves can be affected by various patient-related factors; body characteristics, complex anatomy, or tumor characteristics can affect the learning process [30]. The inclusion of more straightforward and easier cases for initial cases of robot surgery can also lead to an inaccurate learning curve [31]. Further validation studies are needed to arrive at more precise conclusions.

## 5. Conclusions

More experience was required to achieve proficiency for robotic thyroid surgery when complication was used as the marker of proficiency, as opposed to operation time. Incorporation of the operation time, complications, and oncologic outcomes should be considered in learning curve assessment.

## Figures and Tables

**Table 1:** Clinicopathological characteristics of the patients.

Characteristics	Value
Sex (male/female)	19 (11.0%):153 (89.0%)
Age (years)	37.8 ± 9.2 (range, 17–64)
Body mass index (kg/m^2^)	22.6 ± 3.1 (range, 15.9–33.4)
Pathologic characteristics	
Tumor size (cm)	0.8 ± 0.6
Microscopic extrathyroidal extension	98 (57%)
Lymph node metastasis	62 (36.0%)
Number of retrieved lymph nodes	5.4 ± 4.4
Excised thyroid weight (g)	21.0 ± 8.5 (range, 8.2–65.8)
Operation time (min)	142.7 ± 32.1 (range, 69–244)
Complications	
Transient hypoparathyroidism	78 (45.3%)
Transient RLN palsy	9 (5.2%)
Permanent hypoparathyroidism	2 (1.2%)
Permanent RLN palsy	0 (0.0%)
Postoperative bleeding	0 (0%)
Postoperative suppressed thyroglobulin at 3 months	0.18 ± 0.31 (range, 0.00–1.57)
Remnant thyroid tissue on ultrasound at 6 months	0 (0%)

RLN: recurrent laryngeal nerve.

**Table 2:** Comparison of clinicopathological characteristics between the first 50 cases group and the after 51st case group.

Characteristics	The First 50 Cases	After 51st Case	*p*-Value
Sex (male/female)	5:45	14:108	0.779
Age (years)	39.3 ± 7.1	37.1 ± 9.9	0.103
Body mass index (kg/m^2^)	23.1 ± 2.6	22.4 ± 3.3	0.137
Pathologic characteristics			1.000
Tumor size (cm)	0.8 ± 0.4	0.8 ± 0.6	0.477
Microscopic ETE	31 (62.0%)	67 (54.9%)	0.394
LN metastasis	15 (30.0%)	47 (38.5%)	0.290
Number of retrieved LNs	5.3 ± 4.7	5.4 ± 4.3	0.854
Excised thyroid weight (g)	23.0 ± 8.6	20.3 ± 8.4	0.067
Operation time (min)	166.9 ± 29.5	132.8 ± 27.7	<0.001
Complications			
Transient hypoparathyroidism	26 (52.0%)	52 (42.6%)	0.262
Transient RLN palsy	3 (6.0%)	3 (4.9%)	0.772
Permanent hypoparathyroidism	1 (2.0%)	1 (0.8%)	0.512
Permanent RLN palsy	0 (0.0%)	0 (0.0%)	NA
Postoperative bleeding	0 (0.0%)	0 (0.0%)	NA
Suppressed Tg at 3 months	0.22 ± 0.38	0.16 ± 0.28	0.337

ETE: extrathyroidal extension; LN: lymph node; RLN: recurrent laryngeal nerve; Tg: thyroglobulin; NA: not applicable.

**Table 3:** Sensitivity analysis using various cutoff values.

Unacceptable Failure Rate/Acceptable Failure Rate	Decision Limit					
	1.00	1.25	1.50	1.75	2.00	2.25
0.60/0.50	15	72	73	74	75	76
0.60/0.45	14	15	49	73	74	74
0.60/0.40	4	15	15	49	49	73
0.60/0.35	4	4	15	15	49	49
0.55/0.45	49	74	75	76	89	99
0.55/0.40	15	49	73	74	75	75
0.55/0.35	4	15	49	49	73	74
0.50/0.40	73	75	76	100	101	102
0.50/0.35	15	73	74	75	75	76
0.50/0.30	4	15	49	73	74	75
0.45/0.35	74	75	102	156	158	159
0.45/0.30	49	73	74	75	76	157
0.45/0.25	15	49	73	74	75	76
0.40/0.30	75	76	159	160	162	163

## References

[1] Liu S.Y., Kim J.S. (2017). Bilateral axillo-breast approach robotic thyroidectomy: Review of evidences. Gland Surg..

[2] Lee K.E., Rao J., Youn Y.K. (2009). Endoscopic thyroidectomy with the da Vinci robot system using the bilateral axillary breast approach (BABA) technique: Our initial experience. Surg. Laparosc. Endosc. Percutan. Tech..

[3] Koo D.H., Kim D.M., Choi J.Y., Lee K.E., Cho S.H., Youn Y.K. (2015). In-Depth Survey of Scarring and Distress in Patients Undergoing Bilateral Axillo-Breast Approach Robotic Thyroidectomy or Conventional Open Thyroidectomy. Surg. Laparosc. Endosc. Percutan. Tech..

[4] Lee K.E., Koo Do H., Im H.J., Park S.K., Choi J.Y., Paeng J.C., Chung J.K., Oh S.K., Youn Y.K. (2011). Surgical completeness of bilateral axillo-breast approach robotic thyroidectomy: Comparison with conventional open thyroidectomy after propensity score matching. Surgery.

[5] Kwon H., Yi J.W., Song R.Y., Chai Y.J., Kim S.J., Choi J.Y., Lee K.E. (2016). Comparison of Bilateral Axillo-Breast Approach Robotic Thyroidectomy with Open Thyroidectomy for Graves’ Disease. World J. Surg..

[6] Kassite I., Bejan-Angoulvant T., Lardy H., Binet A. (2018). A systematic review of the learning curve in robotic surgery: Range and heterogeneity. Surg. Endosc..

[7] Maruthappu M., El-Harasis M.A., Nagendran M., Orgill D.P., Mcculloch P., Duclos A., Carty M.J. (2014). Systematic review of methodological quality of individual performance measurement in surgery. Br. J. Surg..

[8] Lee J., Yun J.H., Nam K.H., Soh E.Y., Chung W.Y. (2011). The learning curve for robotic thyroidectomy: A multicenter study. Ann. Surg. Oncol..

[9] Kim W.W., Jung J.H., Park H.Y. (2015). A single surgeon’s experience and surgical outcomes of 300 robotic thyroid surgeries using a bilateral axillo-breast approach. J. Surg. Oncol..

[10] Gross N.D. (2012). Commentary: Is robotic thyroid surgery worth the learning curve?. Otolaryngol. Head Neck Surg..

[11] Pernar L.I.M., Robertson F.C., Tavakkoli A., Sheu E.G., Brooks D.C., Smink D.S. (2017). An appraisal of the learning curve in robotic general surgery. Surg. Endosc..

[12] Kwon H., Koo Do H., Choi J.Y., Kim E., Lee K.E., Youn Y.K. (2013). Bilateral axillo-breast approach robotic thyroidectomy for Graves’ disease: An initial experience in a single institute. World J. Surg..

[13] Bokhari M.B., Patel C.B., Ramos-Valadez D.I., Ragupathi M., Haas E.M. (2011). Learning curve for robotic-assisted laparoscopic colorectal surgery. Surg. Endosc..

[14] Yu J., Rao S., Lin Z., Pan Z., Zheng X., Wang Z. (2018). The learning curve of endoscopic thyroid surgery for papillary thyroid microcarcinoma: CUSUM analysis of a single surgeon’s experience. Surg. Endosc..

[15] Biau D.J., Williams S.M., Schlup M.M., Nizard R.S., Porcher R. (2008). Quantitative and individualized assessment of the learning curve using LC-CUSUM. Br. J. Surg..

[16] Segna E., Caruhel J.B., Corre P., Picard A., Biau D., Khonsari R.H. (2018). Quantitative assessment of the learning curve for cleft lip repair using LC-CUSUM. Int. J. Oral Maxillofac. Surg..

[17] Biau D.J., Porcher R. (2010). A method for monitoring a process from an out of control to an in control state: Application to the learning curve. Stat. Med..

[18] Kim M.J., Nam K.H., Lee S.G., Choi J.B., Kim T.H., Lee C.R., Lee J., Kang S.W., Jeong J.J., Chung W.Y. (2018). Yonsei Experience of 5000 Gasless Transaxillary Robotic Thyroidectomies. World J. Surg..

[19] Lee K.E., Kim E., Koo Do H., Choi J.Y., Kim K.H., Youn Y.K. (2013). Robotic thyroidectomy by bilateral axillo-breast approach: Review of 1,026 cases and surgical completeness. Surg. Endosc..

[20] Elliot D.L., Hickam D.H. (1987). Evaluation of physical examination skills. Reliability of faculty observers and patient instructors. JAMA.

[21] Van Rij A.M., Mcdonald J.R., Pettigrew R.A., Putterill M.J., Reddy C.K., Wright J.J. (1995). Cusum as an aid to early assessment of the surgical trainee. Br. J. Surg..

[22] Neuburger J., Walker K., Sherlaw-Johnson C., Van Der Meulen J., Cromwell D.A. (2017). Comparison of control charts for monitoring clinical performance using binary data. BMJ Qual. Saf..

[23] Biau D.J., Resche-Rigon M., Godiris-Petit G., Nizard R.S., Porcher R. (2007). Quality control of surgical and interventional procedures: A review of the CUSUM. Qual. Saf. Health Care.

[24] Edafe O., Antakia R., Laskar N., Uttley L., Balasubramanian S.P. (2014). Systematic review and meta-analysis of predictors of post-thyroidectomy hypocalcaemia. Br. J. Surg..

[25] Anastasiou O.E., Yavropoulou M.P., Papavramidis T.S., Tzouvara C., Triantafyllopoulou K., Papavramidis S., Yovos J.G. (2012). Secretory capacity of the parathyroid glands after total thyroidectomy in normocalcemic subjects. J. Clin. Endocrinol. Metab..

[26] Orloff L.A., Wiseman S.M., Bernet V.J., Fahey T.J., Shaha A.R., Shindo M.L., Snyder S.K., Stack B.C., Sunwoo J.B., Wang M.B. (2018). American Thyroid Association Statement on Postoperative Hypoparathyroidism: Diagnosis, Prevention, and Management in Adults. Thyroid.

[27] Hauch A., Al-Qurayshi Z., Randolph G., Kandil E. (2014). Total thyroidectomy is associated with increased risk of complications for low- and high-volume surgeons. Ann. Surg. Oncol..

[28] Haugen B.R., Alexander E.K., Bible K.C., Doherty G.M., Mandel S.J., Nikiforov Y.E., Pacini F., Randolph G.W., Sawka A.M., Schlumberger M. (2016). 2015 American Thyroid Association Management Guidelines for Adult Patients with Thyroid Nodules and Differentiated Thyroid Cancer: The American Thyroid Association Guidelines Task Force on Thyroid Nodules and Differentiated Thyroid Cancer. Thyroid.

[29] Lee G.S., Arghami A., Dy B.M., Mckenzie T.J., Thompson G.B., Richards M.L. (2016). Robotic single-site adrenalectomy. Surg. Endosc..

[30] Yao A., Iwamoto H., Masago T., Morizane S., Honda M., Sejima T., Takenaka A. (2015). Anatomical dimensions using preoperative magnetic resonance imaging: Impact on the learning curve of robot-assisted laparoscopic prostatectomy. Int. J. Urol..

[31] Bareeq R.A., Jayaraman S., Kiaii B., Schlachta C., Denstedt J.D., Pautler S.E. (2008). The role of surgical simulation and the learning curve in robot-assisted surgery. J. Robot. Surg..

